# High verticality vapor–liquid–solid growth of GaAs_0.99_Bi_0.01_ nanowires using Ga–Bi assisted catalytic droplets†

**DOI:** 10.1039/d3na00428g

**Published:** 2023-11-08

**Authors:** Chalermchai Himwas, Visittapong Yordsri, Chanchana Thanachayanont, Saharat Chomdech, Wenich Pumee, Somsak Panyakeow, Songphol Kanjanachuchai

**Affiliations:** a Semiconductor Device Research Laboratory, Department of Electrical Engineering, Faculty of Engineering, Chulalongkorn University 254 Phayathai Road Bangkok 10330 Thailand Chalermchai.H@chula.ac.th; b National Metal and Materials Technology Center, Thailand Science Park 114 Paholyothin Rd, Klong 1 Klong Luang Pathumthani 12120 Thailand

## Abstract

GaAsBi nanowires (NWs) are promising for optoelectronic applications in the near- and mid-infrared wavelengths due to the optical properties of the Bi-containing compound and the nanowire structure benefits. In general, synthesizing the GaAsBi NWs results in uncontrollable metamorphic structures and spontaneous Bi-containing droplets. Here, we explore the potential of using the droplets as catalysts to form GaAsBi nanowires (hence, the vapor–liquid–solid growth mechanism) on GaAs (111) substrates by molecular beam epitaxy. The GaAsBi NWs experience a two-step growth: Bi droplet deposition and GaAsBi nanowire growth. The optimal droplet deposition temperature (250 °C) is defined based on the droplet morphologies. The gradation of growth temperatures of GaAsBi NWs to 250 °C, 300 °C, and 350 °C results in high-aspect-ratio NWs, tilted NWs, and low-aspect-ratio NWs, respectively. Structural investigation shows that the optimal (low-aspect-ratio) NW has the composition of GaAs_0.99_Bi_0.01_ with the catalytic droplet of Ga_0.99_Bi_0.01_ decorated on its tip. Detailed structural analyses show that the Bi content progressively increases from the NW stem to the wire–substrate interface. The satisfying GaAsBi NW morphology does not warrant the expected superior optical results. Photoluminescence study suggests that the NW has a strong carrier thermalization from the NW stem to the wire–substrate interface influenced by the graded NW growth temperature profile.

## Abstract 2

GaAsBi nanowires (NWs) are promising for optoelectronic applications in the near- and mid-infrared wavelengths due to the optical properties of the Bi-containing compound and the nanowire structure benefits.

## Graphical abstract

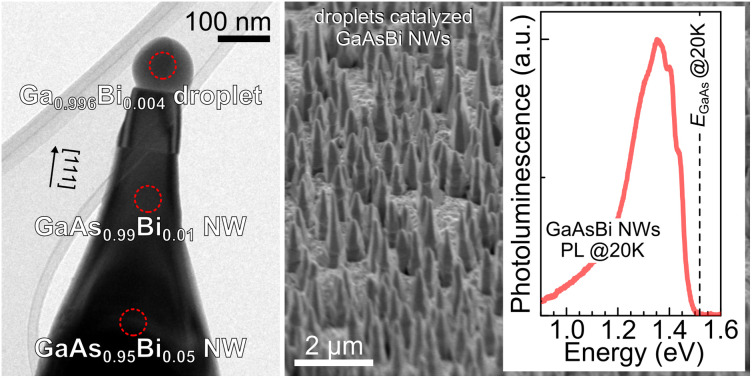

## Introduction

GaAsBi is a relatively new class of III–V semiconductors with many key advantages. Adding Bi into a GaAs matrix can considerably reduce the band gap of the alloy (80–90 meV/Bi%)^1–3^ because GaBi is theoretically a semimetal.^4^ GaAsBi alloys have minimized Auger recombination loss^5,6^ from the energy difference between the valence band and spin–orbit-split-off band which is larger than the band gap. Moreover, the alloy also shows temperature stability.^7^ Despite the advantages, the optimal growth window for GaAsBi is narrow. The growth temperatures and the V/III flux ratios must be well-controlled^8–13^ to incorporate Bi atoms without triggering other challenges during the epitaxial growth, such as phase separation,^14^ surface droplets,^15,16^ and atomic ordering.^17–19^

To enhance the performance of Bi-containing devices, quantum well (QW) or multiple-QW (MQW) structures are often employed since they can improve the optical response through carrier confinement. Most of the Bi-containing devices demonstrated are GaAs_1−*x*_Bi_*x*_ QWs.^20–24^ It proves that low dimensionalities can further extend the performance of semiconductors, and nanowires (NWs) offer possibilities to grow on foreign substrates and to form heterostructures with minimized defect densities.^25–29^ Thus, many research groups have studied the radial and axial heterostructures of GaAs/GaAsBi NWs.^30–33^

However, reports on Bi-containing NWs are limited because of the growth challenges. Ishikawa *et al.* reported GaAs/GaAsBi core–shell NWs grown on Si (111). The GaAsBi shell has the maximum Bi content of 2% and was metamorphically grown around the GaAs core, resulting in a corrugated surface. They attributed the roughness to the lattice mismatch between the GaAs core and GaAsBi shell and the accumulated strain.^30^ The excess Bi atoms accumulated at the hexagonal vertices could act as catalysts for GaAsBi branches along the 〈112〉 directions.^31^ The shell compositional inhomogeneity and metamorphic structure exist even though the strain-balanced GaAs/GaAsPBi core–shell concept is applied.^34^ To obtain homogeneous GaAsBi NWs, one might need to incorporate Bi atoms through a vapor–liquid–solid (VLS) mechanism – the main focus of this work.

Here, we report a two-step technique to synthesize GaAsBi core nanowires on GaAs (111) substrates. The first step revolves around Bi pre-deposition at various growth temperatures/deposition times under an As overpressure to tune for an appropriate Bi amount and density at the growth front. In the second step, the varied growth temperatures and Ga–Bi beam equivalent pressure (BEP) ratios for GaAsBi NWs result in various NW morphologies, *i.e.*, two-dimensional (2D)-like structure, tilted NWs, and vertical NWs. Detailed structural analyses were performed only on the morphology of interest – vertical NWs, confirming that the GaAsBi NWs crystallize by the VLS mechanism *via* Ga–Bi droplets. We briefly report the optical results for passivated GaAsBi core NWs compared to other Bi-containing structures. We associate the severe carrier thermalization with the vertically graded ternary alloy composition, which hinders the luminescence characteristics of the one-dimensional GaAsBi core NWs. Lastly, we suggest the growth conditions, adapted from the experiments, that might result in long-range GaAsBi core NWs with homogeneous compositions.

## Experimental details

Epi-ready GaAs (111) substrates (AXT Inc.) were introduced into an ultra-high-vacuum (UHV) molecular beam epitaxy (MBE) system. The substrates were outgassed in the preheat chamber at 400 °C for 1 hour and were subsequently de-oxidized in the growth chamber at 600 °C for 30 min under an As-overpressure (BEP_As_ = 8 × 10^−6^ torr). The outgassed and de-oxidized processes expelled contaminants and oxides from the surfaces. The former outgassing in the preheat chamber minimized the introduction of possible contaminants into the growth chamber. The de-oxidation performed at the higher temperature and under an As-overpressure was to compensate for the sublimated As atoms from the surfaces. The substrate temperatures (*T*_s_) were derived from a non-contact thermocouple temperature calibrated against the de-oxidation reflection high-energy electron diffraction (RHEED) pattern known to occur at 580 °C.

After the de-oxidation process, Bi droplets were deposited on the GaAs (111) substrates. The deposition was performed by opening the Bi shutter (at BEP_Bi_ = 5 × 10^−8^ torr) under a constant As flux (BEP_As_ = 3.0 × 10^−6^ torr). The Bi droplets were deposited at various substrate temperatures (*T*_s_ = 200 (sample D1), 250 (D2, D5, and D6), 275 (D3), and 300 °C (D4)) with specific droplet deposition times (*t*_d,D_). Table 1 presents the growth parameters and morphologies of the Bi droplets under study. The Bi deposition was performed in the *T*_s_ range of 200–300 °C under an As overpressure. The choice of the *T*_s_ range is made similar to those of Bi-containing materials while the BEP_As_ value was predetermined identical to the following growth sequence. Since our As flux is supplied by a sublimation cell, we cannot abruptly switch the BEP_As_ – the crucial parameter for fostering NWs. To overcome this challenge, we set the desired value of BEP_As_ before the deposition of Bi droplets and kept it constant until the NW growth was complete.

**Table 1:** Description of the Bi droplet deposition conditions on GaAs (111) substrates. Key deposition parameters: substrate temperature (*T*_s_), droplet deposition time (*t*_d,D_), As, and Bi beam equivalent pressures are BEP_As_ = 3.0 × 10^−6^ torr, and BEP_Bi_ = 5 × 10^−8^ torr. Extracted droplet morphologies: droplet density, droplet diameter, and droplet height

Sample	Bi droplet deposition conditions		Droplet density (μm^−2^)	Diameter (nm)	Height (nm)
	*T*_s_ (°C)	*t*_d,D_ (min)			
D1	200	2	42	45.6 ± 3.2	9.6 ± 1.6
D2	250	10	1.81	307.2 ± 80.0	70.2 ± 27.9
D3	275	10	0.28	250.7 ± 79.4	96.8 ± 33.9
D4	300	25	—	—	—
D5	250	2	1.89	190.6 ± 62.5	25.0 ± 14.8
D6	250	20	1.52	353.8 ± 91.9	76.0 ± 33.2

After the droplet deposition, the NWs were initiated by opening the Ga shutter at BEP_Ga_ = 3 × 10^−7^ torr, equivalent to a 2D deposition rate on GaAs (001) *R*_GaAs_ of 0.57 nm s^−1^ as calibrated by RHEED oscillations. The BEP_As_ and BEP_Bi_ were kept identical to the previous stage. The GaAsBi NWs were grown with different substrate temperature ramping profiles. Samples NW1, NW2, and NW3 were grown during the temperature ramping from 250 °C to 250 °C (*i.e.*, no ramping), 300 °C, and 350 °C, respectively. To explore the effect of the NW deposition time (*t*_d,NW_), we also synthesized NW4, whose growth conditions are identical to NW3 except for the extended *t*_d,NW_ = 30 min.

For the optical study, we exclusively synthesized NW5. The sample experienced two more growth steps: droplet crystallization; and GaP shell radial growth. Because the GaAs surface has high surface recombination velocity^35,36^ and acts as non-radiative recombination centers, passivation with a higher band gap material (GaP) facilitates luminescence studies. For NW5, the NW growth conditions are identical to those for NW3/NW4, except for *t*_d,NW_ = 20 min. The droplet crystallization was performed by exposing GaAsBi NWs to only As flux (closing Ga and Bi shutters) for 15 min while keeping the *T*_s_ at 350 °C. After the droplet crystallization, *T*_s_, BEP_Ga_, and BEP_P2_ were set to 450 °C, 3.3 × 10^−8^ torr, and 6.6 × 10^−7^ torr, and then the P shutter (As shutter) was opened (closed). Growth interruption after the P shutter opening was carried out until the growth chamber pressure stabilized (∼10 min) to ensure the P overpressure. The GaP passivation was performed by opening the Ga shutter for 2 min 30 s. Table 2 presents the growth parameters and structural properties of the GaAsBi NW samples under study.

**Table 2:** Description of the GaAsBi nanowire deposition conditions. Key deposition parameters: substrate temperature (*T*_s_), nanowire deposition time (*t*_d,NW_), Ga, As, and Bi beam equivalent pressures (BEP) are BEP_Ga_ = 3.0 × 10^−7^ torr, BEP_As_ = 3.0 × 10^−6^ torr, and BEP_Bi_ = 5 × 10^−8^ torr. Extracted nanowire morphologies: nanowire bottom diameter, top diameter, and nanowire height

Sample	GaAsBi NW deposition conditions		Bottom diameter (nm)	Top diameter (nm)	Height (μm)
	*T*_s_ (°C)	*t*_d,NW_ (min)			
NW1	250	6	283	268	0.47
NW2	300	7	350	77	0.33
NW3	350	6	256	113	0.49
NW4	350	30	567	180	1.49
NW5	350	20	388	212	0.85

The morphologies of the droplets were explored using tapping mode atomic force microscopy (AFM), while the structural information of the NWs was acquired using scanning electron microscopy (SEM). For detailed structural analyses, selected NWs were transferred onto a holey-carbon–copper grid. The NW structure and composition were analyzed by transmission electron microscopy (TEM, JEOL JEM-2010) equipped with energy-dispersed X-ray spectroscopy (EDS, OXFORD). Selected area diffraction patterns (SADPs) were recorded for crystallographic identification. TEM-EDS was probed at specific areas for elemental characterization. Optical characterization was performed by recording the photoluminescence (PL) spectra. Steady-state PL spectra were obtained by exciting the NWs with a Ventus solid-state laser (*λ* = 532 nm) and collecting the emission with a Horiba iHR320 monochromator equipped with a cooled (77 K) InGaAs photodetector.

## Results and discussion

### Bi droplets

During the Bi deposition, RHEED patterns changed from the GaAs (111) diffraction pattern to a dim reflection, confirming the Bi deposition. The RHEED pattern evolutions for samples D1, D3, D5, and D6 are provided in ESI S1.†

**Fig. 1:**
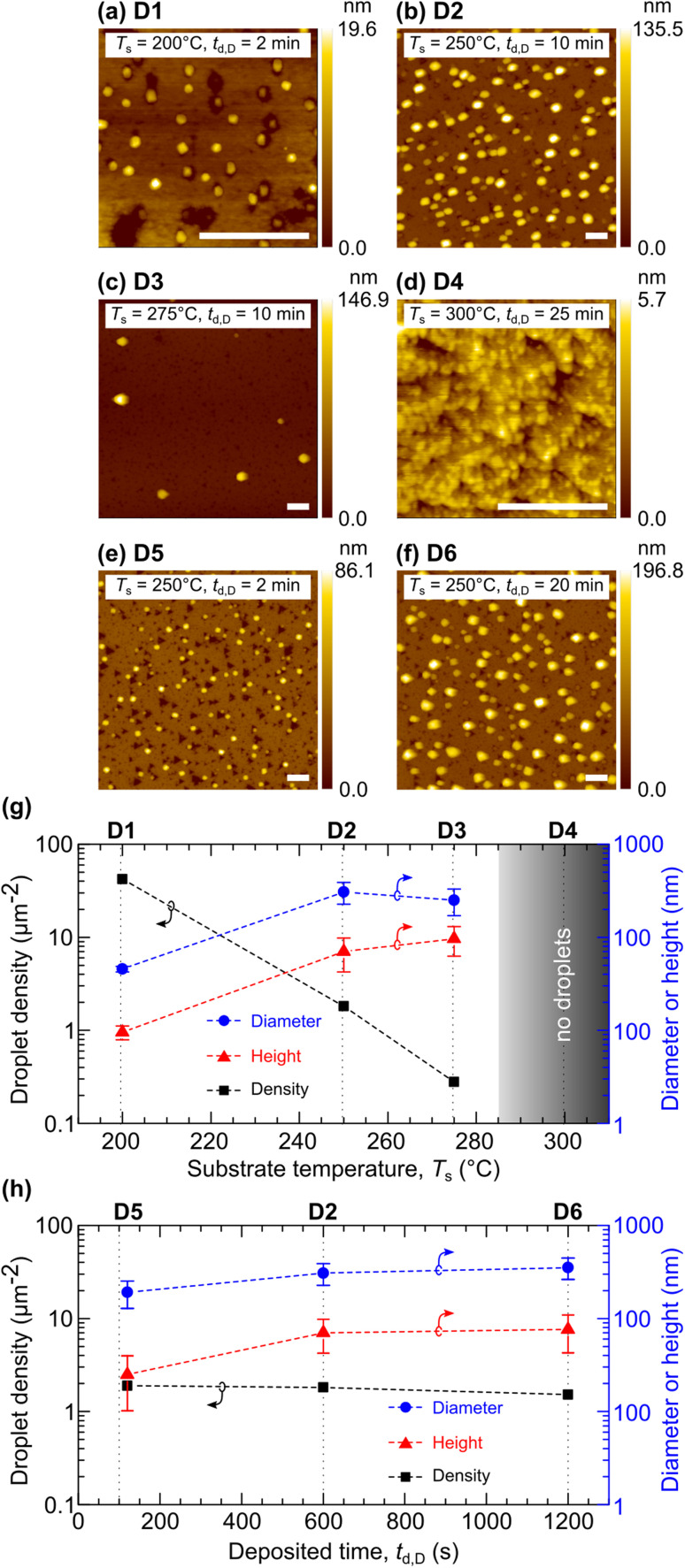
(a)–(f) AFM images of Bi droplets synthesized at various substrate temperatures (*T*_s_) and deposition times (*t*_d,D_). Scale bars in (a)–(f) correspond to 0.5 μm. (g) Variations of droplet heights, diameters, and droplet densities as a function of *T*_s_ for samples D1, D2, and D3. (h) Variations of droplet heights, diameters, and droplet densities as a function of *t*_d,D_ for samples D2, D5, and D6.

Fig. 1(a)–(f) are the AFM images of samples D1–D6 depicting the Bi droplet morphologies. Their morphological parameters (diameter, height, and density) were extracted and are plotted in Fig. 1(g) and (h). Fig. 1(g) shows the variations of the morphological parameters as a function of *T*_s_. Droplet diameters (droplet heights) for D1, D2 and D3 are 45.6 ± 3.2 (9.6 ± 1.6), 307.2 ± 80.0 (70.2 ± 27.9), and 250.7 ± 79.4 (96.8 ± 33.9) nm, respectively. The error bars represent the standard deviation of uncertainty. The distinct *t*_d,D_ value for D1 hinders the morphological comparison with the other two samples. The variations for D2 and D3 suggest that the deposition at higher *T*_s_ (with identical *t*_d,D_) results in a lower droplet aspect ratio. The aspect ratio (defined as the ratio between the diameter and height) for D2 is 4.37, and that for D3 is 2.59. Despite the distinct *t*_d,D_ for D1, Fig. 1(g) shows that the Bi droplet density exponentially decreases with increasing *T*_s_ (from 42 droplets per μm^2^ at *T*_s_ = 200 °C to 0 droplets per μm^2^ at *T*_s_ = 300 °C). Baraissov *et al.* studied Ga metallic droplets deposited at various substrate temperatures and observed the same trend.^37^Fig. 1(h) shows the variations of the morphological parameters as a function of *t*_d,D_ with the identical *T*_s_ = 250 °C. Droplet diameters (droplet heights) for D5, D2, and D6 are 190.6 ± 62.5 (25.0 ± 14.8), 307.2 ± 80.0 (70.2 ± 27.9), and 353.8 ± 91.9 (76.0 ± 33.2) nm, respectively. The Bi droplet densities for D5, D2, and D6 are similar in the 1.52–1.89 droplets per μm^2^ range. These variations suggest that the droplet density varies consistently with *T*_s_ (Fig. 1(g)) while the *t*_d,D_ (Fig. 1(h)) controls the diameter and height.

Based on the results, one should deposit Bi droplets at *T*_s_ = 200–250 °C (below the Bi melting point at 271.4 °C) for a reasonable Bi droplet density. We chose to deposit the Bi droplets at *T*_s_ = 250 °C for *t*_d,D_ = 10 min, which results in 1.81 droplets per μm^2^ to proceed with the subsequent step of GaAsBi NW growth.

### GaAs_1−*x*_Bi_*x*_ nanowires

On this topic, we report the morphologies of GaAs_1−*x*_Bi_*x*_ NWs grown at different *T*_s_. NWs of interest were selected for detailed structural studies. At the end of this topic, we primitively discuss the luminescence behavior of GaAs_1−*x*_Bi_*x*_ NWs.

**Fig. 2:**
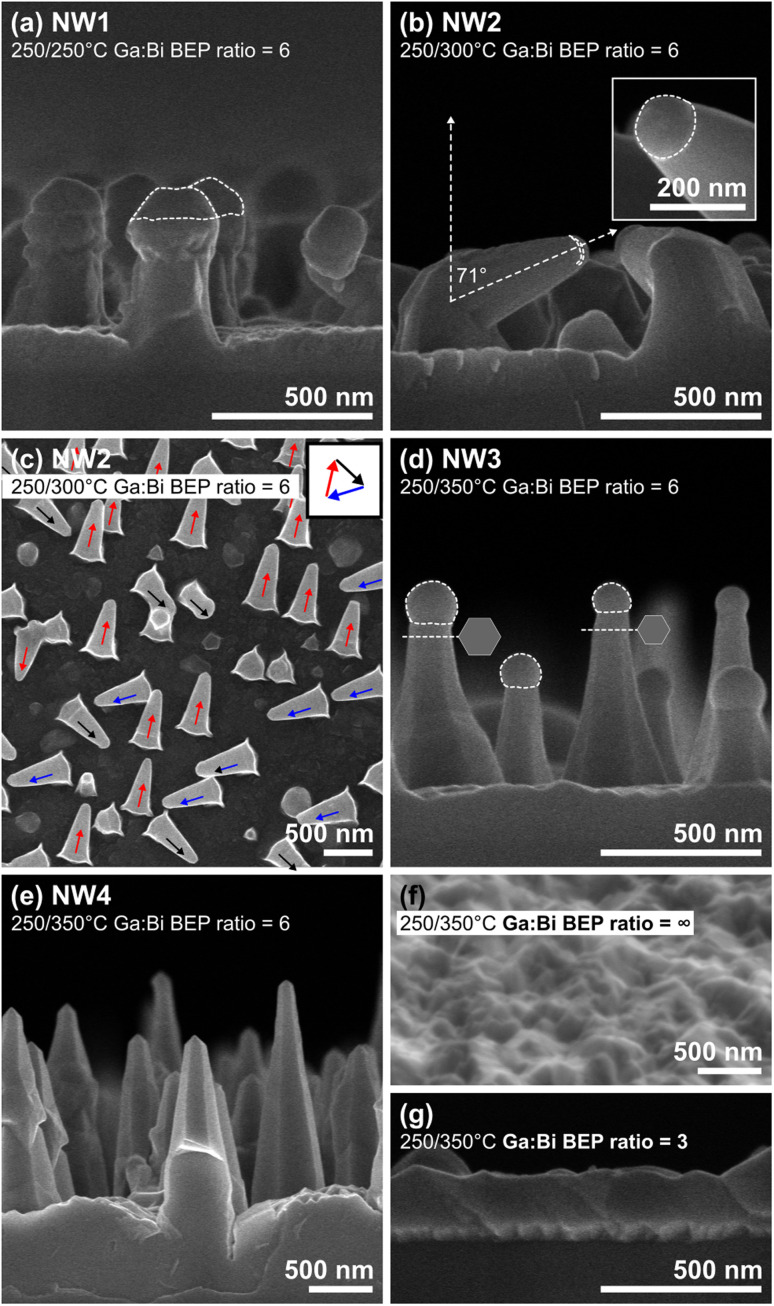
(a), (b), (d) and (e) are 90°-tilted SEM images of GaAsBi nanowires grown by ramping *T*_s_ from 250 °C to 250 °C (sample NW1) to 300 °C (NW2), 350 °C (NW3), and 350 °C (NW4), respectively. (c) Top-view SEM image of NW2 depicted NW tilted directions. (f) 10°-tilted and (g) 90°-tilted SEM images consecutively show the surfaces grown using the NW4 growth conditions without supplying Bi flux and with a Ga-to-Bi beam equivalent pressure ratio of 3. Scale bars in (a)–(g) correspond to 500 nm, and that in the inset of (b) corresponds to 200 nm.

Fig. 2(a) shows a 90°-tiled SEM image of GaAsBi NWs with catalytic droplets on top (see white dashed peripheries). The average height of NW1 is 0.47 μm above the parasitic growth, and the average diameter is 275 nm. The wires grown with the identical *T*_s_ to that of Bi droplet deposition seem to have metamorphic structures yet develop along the [111] axis. Because the NW growth temperature is lower than the Bi melting point (∼271.4 °C), the liquid droplets that assisted the NW growth could be a Ga–Bi binary compound since its melting point decreases from that of the pure Bi case. NW2 has a tapered shape and a distinct orientation shown in Fig. 2(b) when the NW growth was performed simultaneously with the temperature increasing from 250 °C to 300 °C. NW2 has an average base diameter of 350 nm and an average height of 0.25 μm before switching its orientation. It is believed that the tilting occurred owing to wetting of the droplets at one of the NW facets during the growth temperature modification from a graded temperature to the fixed *T*_s_ = 300 °C, a mechanism similar to the effect of *in situ* annealing applied to control the GaAs NW direction,^38^ resulting in three possible directions as demonstrated in the top-view SEM image of sample NW2 (Fig. 2(c)). The tilting occurred only for NW2 with an angle of 71° normal to the surface. After the growth proceeds in a new direction, the NW diameter underneath the catalytic droplet is 143 nm. NW3 (NW4) has an average height of 0.49 μm (1.49 μm) above the parasitic growth, an average base diameter of 256 nm (567 nm), and an average top diameter of 113 nm (180 nm). Both samples have a 100% vertical yield, as depicted in Fig. 2(d) and (e). Catalytic droplets are evidenced for NW3 (white dashed peripheries), but no droplets were found for NW4. The droplets that disappear when increasing *t*_d,NW_ from 6 min (NW3) to 30 min (NW4) evolve similar to the Ga droplet-assisted VLS mechanism reported elsewhere.^39,40^ The growth mechanism switching and the pyramidal shape suggest that the ternary alloy NWs evolved under As-rich conditions. Indeed, we initially expected hexagonal prism GaAsBi NWs with the base diameter similar to the top diameter because the BEP_As_/BEP_Ga_ value is 10, which is close to stoichiometric condition for the general growth of GaAs NWs by MBE. Here, the syntheses at comparatively lower temperatures decrease the As desorption from the growth fronts, resulting in more As atoms participating in the wire growth. To obtain the hexagonal prism shape for GaAsBi NWs grown at *T*_s_ = 350 °C, one needs to modify the BEP_As_/BEP_Ga_ ratio. Furthermore, we proved that the amount of Bi atoms is critical for fostering the GaAsBi NWs at this low growth temperature. While all the NW samples were grown using BEP_Ga_/BEP_Bi_ = 6, the micrographs for the growth without supplying Bi flux (BEP_Ga_/BEP_Bi_ = ∞) and with BEP_Ga_/BEP_Bi_ = 3 are consecutively demonstrated in Fig. 2(f) and (g), resulting only in the 2D-parasitic deposition.

**Fig. 3:**
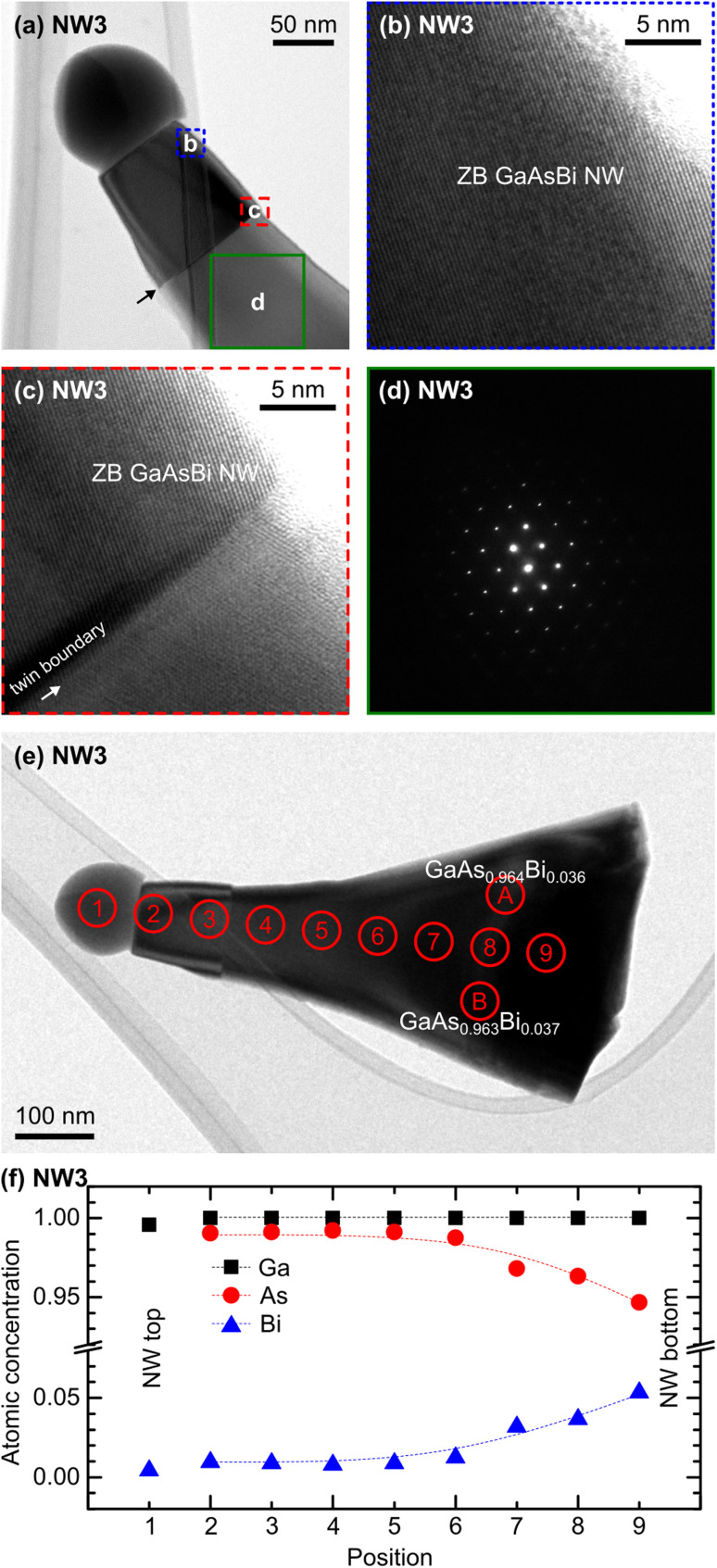
(a) BF-TEM micrograph of sample NW3 oriented along the [111] direction shows one twin plane boundary. (b) and (c) High-resolution TEM micrographs recorded at the areas marked in (a), revealing the ZB crystal structure for both regions with one twin boundary. (d) Diffraction pattern recorded over the large area on the NW marked in (a), revealing the pure crystal phase (ZB) over the large area. (e) BF-TEM micrograph of sample NW3 with delimited areas ‘1’, ‘2’, ‘3’, ‘4’, ‘5’, ‘6’, ‘7’, ‘8’, ‘9’, ‘A’, and ‘B’, showing the areas where EDS acquisitions were performed. (f) Corresponding Ga, As, and Bi atomic concentrations at each area delimited in (e), portraying the vertical and cross-sectional variations of Ga, As, and Bi concentrations.

Fig. 3(a) is a bright-field (BF-) TEM micrograph for NW3. The contrast shift (black arrow) at the NW middle suggests the crystal structure alteration. Individual analyses of NW3 show that the wire has a tapered shape with a base diameter of 324 nm and a top diameter of 91 nm. Fig. 3(b) and (c) are high-resolution (HR-) BF-TEM micrographs recorded at the ‘blue square’ and ‘red square’ regions delimited in Fig. 3(a), respectively. HR-BF-TEM micrographs confirm that the NW crystallized with a zinc blende structure with a single twin boundary (white arrow in Fig. 3(c)) over the entire NW length. Indeed, this is the only twin boundary found from several nanowires (NW3) submitted to detailed structural analyses. We also performed SADP over the selected area delimited by the ‘green square’ in Fig. 3(a). As shown in Fig. 3(d), the clear diffraction pattern implies a pure crystal phase over the large acquisition area.

TEM-EDS measurements were performed at various positions on the wire (see Fig. 3(e)), starting from the catalytic droplet marked as ‘1’ down to the NW bottom marked as ‘9’. Their corresponding Ga, As, and Bi atomic concentrations are plotted in Fig. 3(f). The alloy composition at the catalytic droplet (area ‘1’) is Ga_0.996_Bi_0.004_. Note that the ‘As-free’ at the droplet is crucial for this context as it proves that NW3 (also NW1 and NW2, where the catalytic droplets are shown earlier) evolved by the VLS mechanism catalyzed by the spherical liquid droplets. The ternary alloy compositions are stable from the area ‘2’ to ‘5’ at GaAs_0.99_Bi_0.01_. To the NW bottom, the As content (Bi content) continually decreases (increases) and reaches its minimum (maximum) at area ‘9’. The composition in this area is GaAs_0.95_Bi_0.05_. This compositional modification is associated with the temperature increment from 250 °C to 350 °C during the NW growth. The wire at the substrate proximity grown at 250 °C has a higher Bi content than the NW top section grown at 350 °C.

Cross-sectionally, the ternary alloy compositions probed at the areas ‘A’ and ‘B’ (see Fig. 3(e)) are GaAs_0.964_Bi_0.036_ and GaAs_0.963_Bi_0.387_, respectively. The similar contents confirm the benefit of the VLS mechanism, resulting in evenly distributed Bi atoms across the NW diameter and along the NW length (at a constant *T*_s_), minimizing alloy inhomogeneity – the main challenge when incorporating Bi atoms in nanostructures. Detailed TEM-EDS compositional measurements are provided in ESI S2.†

**Fig. 4:**
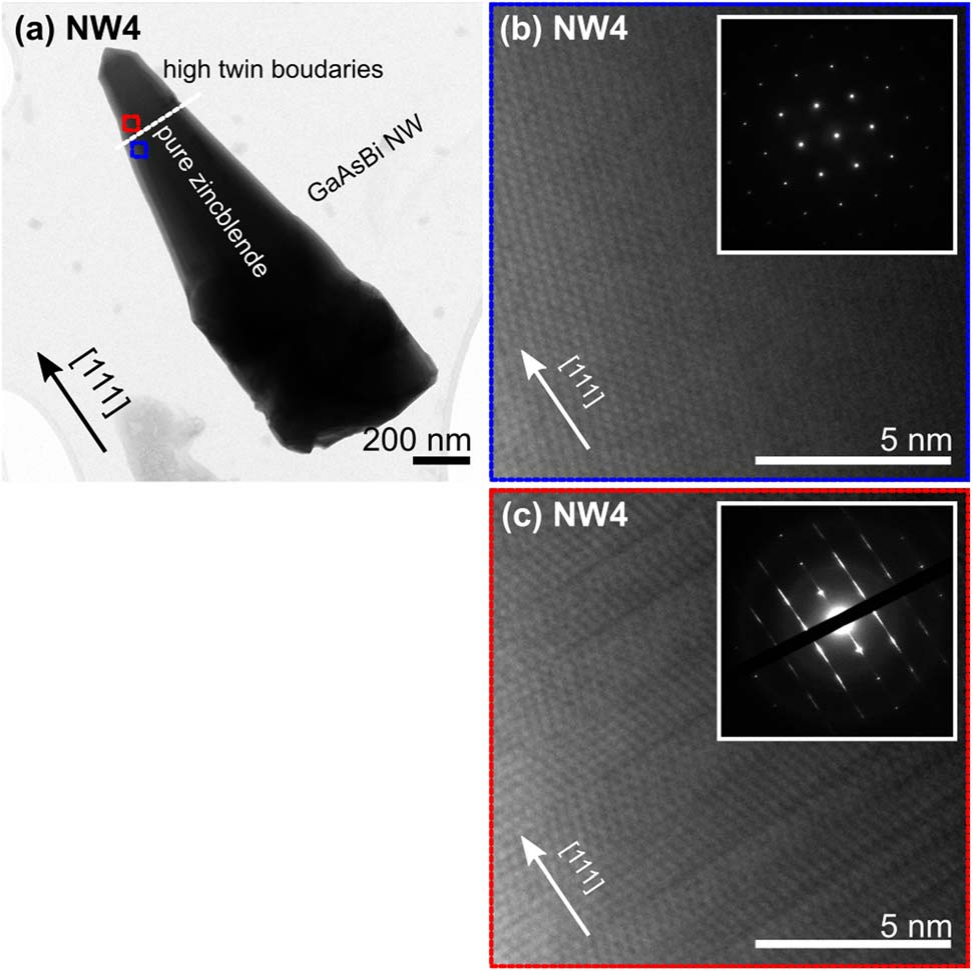
(a) BF-TEM micrograph of sample NW4 oriented along the [111] direction shows the pure zinc blende section and the section with high twin boundaries on the NW tip. (b) High-resolution TEM micrographs were recorded at the NW stem and the NW tip regions, the marked areas with the blue-square and red-square in (c). The insets for (b) and (c) show their corresponding SADPs, revealing the pure crystal phase at the NW stem and the mixed phase at the NW tip. The scale bar in (a) corresponds to 200 nm, and those in (b) and (c) correspond to 5 nm.

Fig. 4(a) is a BF-TEM micrograph for NW4. Individual analyses of NW4 show that the wire has a tapered shape with a base diameter of 507 nm and a top diameter of 214 nm. The monotonous contrast at the NW stem and the alternating contrast at the NW tip in Fig. 4(a) suggest that the NW crystallized with different crystal phases at the two regions. HR-BF-TEM micrographs were performed at the ‘blue square’ and ‘red square’ regions to explore the crystal phase. The micrograph of the NW stem (NW4) oriented along the [111] direction and the corresponding SADP (Fig. 4(b)) reveal that the NW stem crystallized with the zinc blende (ZB) structure over the NW length. The micrograph of the NW and the corresponding SADP (Fig. 4(c)) reveal that the NW tip crystallized with a high density of twin planes normal to the growth axis. The excess As flux condition shirked the catalytic droplets until they were entirely crystallized for NW4 (the sample grown with a comparatively higher *t*_d,NW_ value). After the complete consumption of the droplets, the NW growth switched to the vapor–solid mechanism, which could result in a core–shell structure. The distinct compositions of the core and shell can disturb the compositional estimation. Thus, we only report the ternary alloy composition of NW3 where the catalytic droplets still exist, ensuring that the probed value belongs to the “core only” GaAsBi NW.

**Fig. 5:**
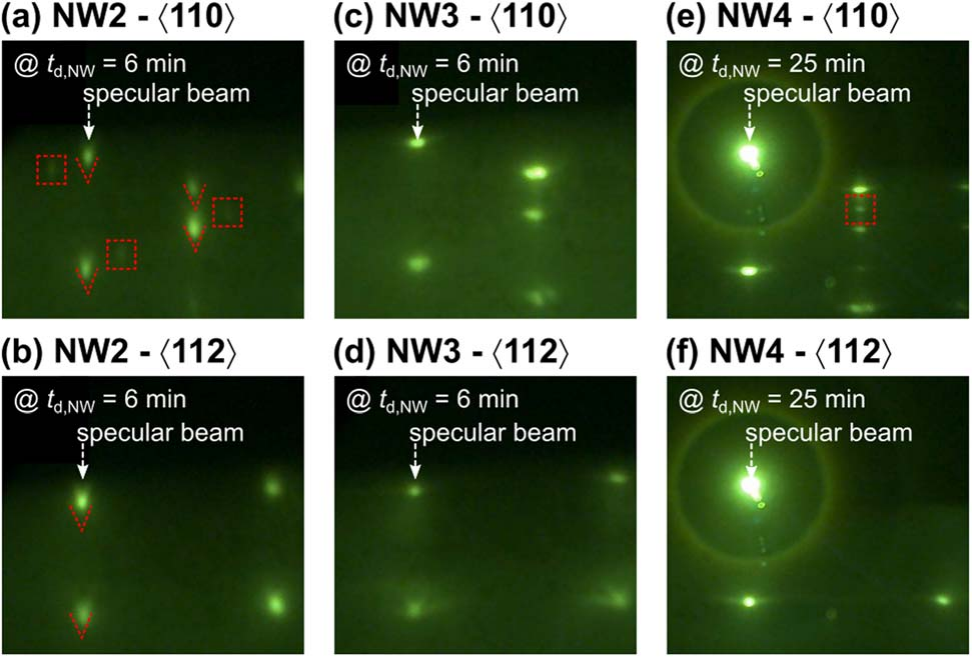
(a)–(f) RHEED diffraction patterns recorded along the 〈110〉 and 〈112〉 azimuths during the growth of NW2, NW3, and NW4. The images were snapped at different GaAsBi nanowire deposition times (*t*_d,NW_) as provided in each panel.

*In situ* RHEED patterns along the 〈110〉 and 〈112〉 azimuths for samples NW2, NW3, and NW4 are discussed against the reported morphologies. The RHEED diffraction intensity for NW1 dims (unshown), and various diffraction spots occur, possibly due to the large volume of amorphous liquid droplets on the growth front and a mixing structure during the NW initiation. Fig. 5(a) and (b) show the RHEED patterns of NW2 recorded along the 〈110〉 and 〈112〉 azimuths at *t*_d,NW_ = 6 min. More diffraction spots emerge along the 〈110〉 azimuth marked in Fig. 5(a) with red dashed squares. Typical diffraction spots along the 〈110〉 and 〈112〉 azimuths resulting from ZB NWs transform into chevrons as guided to eyes in Fig. 5(a) and (b) by red dashed chevrons. The emerged diffraction spots and diffraction transformation occurred only for NW2, which is associated with the tilted NWs. Fig. 5(c) and (d) show the RHEED diffraction patterns of NW3 recorded along the 〈110〉 and 〈112〉 azimuths at *t*_d,NW_ = 6 min. The NW deposition time is sufficiently short to preserve the catalytic droplets on the wire tips under the As-rich growth condition but long enough for the NWs to vertically evolve with the pure ZB crystal structure as confirmed *in situ* by the diffraction patterns. Fig. 5(e) and (f) show the RHEED diffraction patterns of NW4 recorded along the 〈110〉 and 〈112〉 azimuths at *t*_d,NW_ = 25 min. To the NW tips, the growth evolves with progressively lower droplet contact angle under As-rich conditions introducing a wurtzite (WZ) structure and polytypic phase before the droplets are entirely crystallized. According to the sequence, one should observe the WZ spots before the droplet is successfully consumed. The diffraction spot related to the WZ phase emerges after growing the NWs for more than 20 min (as marked with a red dashed square in Fig. 5(e)) overlayed on the diffraction pattern of the ZB structure, suggesting that the top part of NW4 contains both ZB and WZ structures, in agreement with the HR-TEM results (Fig. 4(a) and (c)). The RHEED patterns of GaAs NWs recorded along the 〈110〉 azimuth superposed with an indexation diagram can be found elsewhere for comparison.^41^

To study the luminescence of GaAs_0.99_Bi_0.01_ NWs, NW5 was exclusively synthesized by adapting from NW4 growth conditions. Droplets of NW5 were crystallized, and the GaAsBi cores were surrounded by thin GaP shells. Passivation with a higher band gap material facilitates luminescence studies^34,42,43^ since the GaAs surface has high surface recombination velocity^35,36^ and acts as a non-radiative recombination center.

**Fig. 6:**
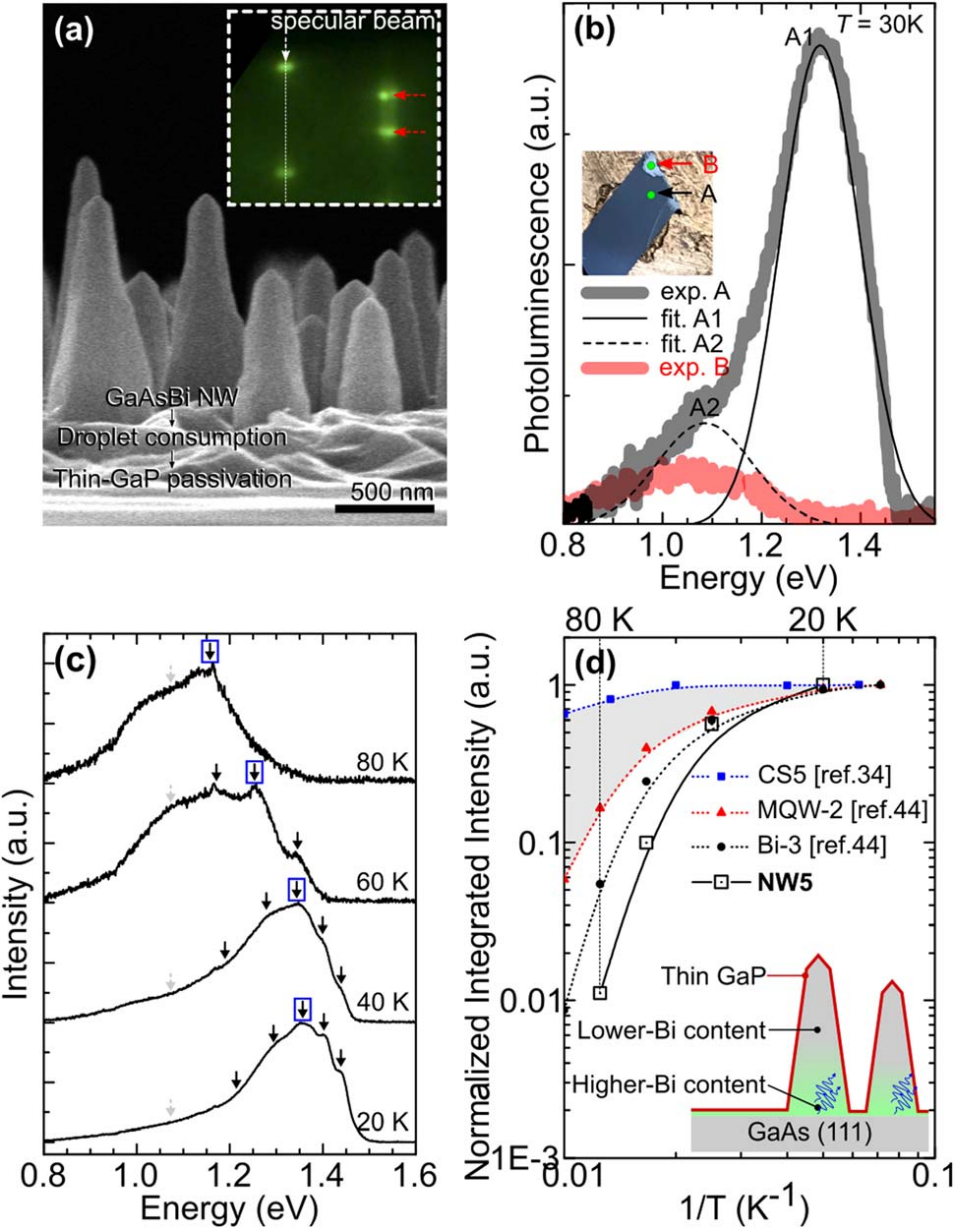
(a) 90°-Tilted SEM image of GaP-passivated GaAsBi nanowires (NW5). The inset shows the RHEED diffraction pattern along the 〈110〉 azimuth during the NW growth. (b) PL emission spectra of NW5 measured at 30 K. Black and red spectra are acquired from the as-grown area and NW-scraped-out area, respectively. (c) Temperature-dependent PL spectra measured from 20 K to 80 K. (d) Integrated PL intensity as a function of inverse temperature for NW5. For comparison, the PL peak evolutions for Bi-containing nanowires (blue squares, ref. 34), multiple quantum wells (red triangles, ref. 44), and bulk (black circles, ref. 44), are added in panel (d).

Fig. 6(a) depicts the SEM image of NW5. Note that the RHEED patterns along the 〈110〉 azimuth during the NW growth step, droplet crystallization step, and passivation step are similar to those shown in the inset of Fig. 6(a) and identical to those of NW3 (Fig. 5(b)). It suggests that NW5 has a lower density (if any) of WZ or polytypic structures than NW4. Fig. 6(b) shows the photoluminescence spectra for NW5 measured at 30 K. Luminescence spectra ‘A’ and ‘B’ arise from the as-grown and the NW-scraped-out areas, respectively. Top-view SEM images for both positions are provided in ESI S3,† showing that the density for NW5 is 1.22 wire per μm^2^. The NW5 density is similar to the droplet density initiated at *T*_s_ = 250 °C (see Fig. 1(g) and (h)), suggesting that these wires emerged from the previously deposited droplets. The slight discrepancy is associated with the fact that some droplets do not evolve into NWs. Short NWs were not seen from the top-view SEM micrograph, hence excluded from the NW statistical analysis.

Macroscopically, one can visually distinguish the two areas demonstrated in the inset of Fig. 6(b), where the green dots represent the laser spots during the luminescence acquisitions. The spectrum ‘A’ is fitted into two Gaussian peaks at 1.32 eV (peak A1) and 1.08 eV (peak A2), while the spectrum ‘B’ shows only one Gaussian peak at ∼1.1 eV. Since most of the wires were scraped out from ‘B’, leading to the formation of the two-dimensional parasitic material before the NW growth, the luminescence peak at ∼1.1 eV is associated with the parasitic growth on the surface. Henceforth, we exclude the luminescence peak at ∼1.1 eV from our GaAsBi NW luminescence discussion. Fig. 6(c) shows normalized PL spectra for NW5 measured at 20, 40, 60, and 80 K. GaAsBi NW luminescence spectra fitted into many Gaussians. We exclude the peaks marked with gray-dashed arrows (∼1.1 eV) from our optical analyses.

Fig. 6(d) shows the integrated PL intensity as a function of inverse temperature normalized to its value at 20 K. The integrated PL intensity drops very quickly. Comparing the normalized integrated intensity at 80 K (*I*_80_) against that at 20 K (*I*_20_), *I*_80_/*I*_20_ is 0.011. To further understand, we compare the result with other Bi-containing structures: bulk,^44^ multiple quantum wells,^44^ and nanowires.^34^ By the same criteria, *I*_80_/*I*_20_ values for Bi containing bulk, multiple-quantum wells, and nanowires are 0.05, 0.15, and 0.8, respectively. Theoretically, the NW should possess two-dimensional confinement behavior. Thus, we expected the *I*_80_/*I*_20_ to be higher than the one-dimensional confinement multiple quantum wells (*I*_80_/*I*_20_ = 0.15). Its integrated PL intensity evolution as a function of inverse temperature should lie in the shaded area in Fig. 6(d). In our case, the *I*_80_/*I*_20_ value for NW5 is even lower than that of Bi3 (bulk). It is because the generated carriers in the wire transfer to a band gap minima region (wire–substrate interface) at elevated temperatures before recombining (see the schematic demonstrated in Fig. 6(d)). The lower (higher) Bi concentration at the NW top (wire–substrate interface) as structurally evidenced in Fig. 3(e) and (f) creates the carrier thermalization channel that prohibits us from exploring the genuine optical behavior of one-dimensional GaAsBi NWs.

## Conclusion

We have synthesized GaAsBi NWs by the VLS mechanism catalyzed by Ga–Bi droplets. The NWs have grown on GaAs (111) substrates by MBE using a two-step growth technique (Bi droplet predeposition and GaAsBi NW growth). We deposited Bi droplets for 10 min at the substrate temperature of 250 °C to obtain the suitable droplet morphologies to catalyze the NWs. By varying the graded NW growth temperatures from 250 °C to 250 °C, 300 °C, and 350 °C, the NW morphologies consecutively change to high-aspect-ratio NWs along the [111] axis, tilted NWs, and low-aspect-ratio NWs along the [111] axis respectively. Based on the high vertical yield and compositional information, we obtain the optimal GaAsBi NWs at the substrate temperature of 350 °C. Detailed structural analyses of the optimal NWs show that GaAsBi NWs crystallized with a pure ZB structure with the catalytic droplets on the NW tips. The graded NW growth temperature to 350 °C resulted in the homogeneous composition across the NW diameter and progressively lower Bi content from the bottom to the top of the NW, ending with a Ga_0.99_Bi_0.01_ droplet. The highest Bi content (lowest Bi content) probed at the NW/substrate interface (at the NW middle) is 0.05 (0.01). The photoluminescence peak measured at 20 K is 1.35 eV. However, we found that the wire has a strong carrier thermalization toward the wire–substrate interface supported by the structural results, which hinders the exploration of the wire's one-dimensional behavior. One may consider adjusting the V-to-III flux ratio or substrate temperature during the GaAsBi NW growth to minimize the carrier thermalization effect.

## Author contributions

Chalermchai Himwas: conceptualization, methodology, investigation, writing – original draft, writing – review & editing. Visittapong Yordsri: investigation. Chanchana Thanachayanont: investigation. Wenich Pumee: investigation. Somsak Panyakeow: resources. Songphol Kanjanachuchai: resources.

## Conflicts of interest

There are no conflicts to declare.

## Supplementary Material

NA-006-D3NA00428G-s001
