# Supplementary material for: High verticality vapor–liquid–solid growth of GaAs0.99Bi0.01 nanowires using Ga–Bi assisted catalytic droplets
Source: Nanoscale Adv. 2023 Nov 8;6(3):846–54. doi: 10.1039/d3na00428g (PMC10825910; doi:10.1039/d3na00428g)
Supplement: NA-006-D3NA00428G-s001 [file NA-006-D3NA00428G-s001.pdf]

Electronic Supplementary Material (ESI) for Nanoscale Advances.
This journal is © The Royal Society of Chemistry 2023

Supplementary Information

High verticality vapor-liquid-solid growths of $GaAs_{0.99}Bi_{0.01}$ nanowires using Ga-Bi assisted catalytic droplets.

Chalermchai Himwas,[*a] Visittapong Yordsri,[b] Chanchana Thanachayanont,[b] Saharat Chomdech,[a] Wenich Pumee,[a] Somsak Panyakeow,[a] and Songphol Kanjanachuchai[a]

[a]*Semiconductor Device Research Laboratory, Department of Electrical Engineering, Faculty of Engineering, Chulalongkorn University, 254 Phayathai Road, Bangkok 10330, Thailand*

[b]*National Metal and Materials Technology Center, Thailand Science Park, 114 Paholyothin Rd., Klong 1, Klong Luang, Pathumthani 12120, Thailand*

S1: The RHEED diffraction patterns for samples D1, D3, D5, and D6

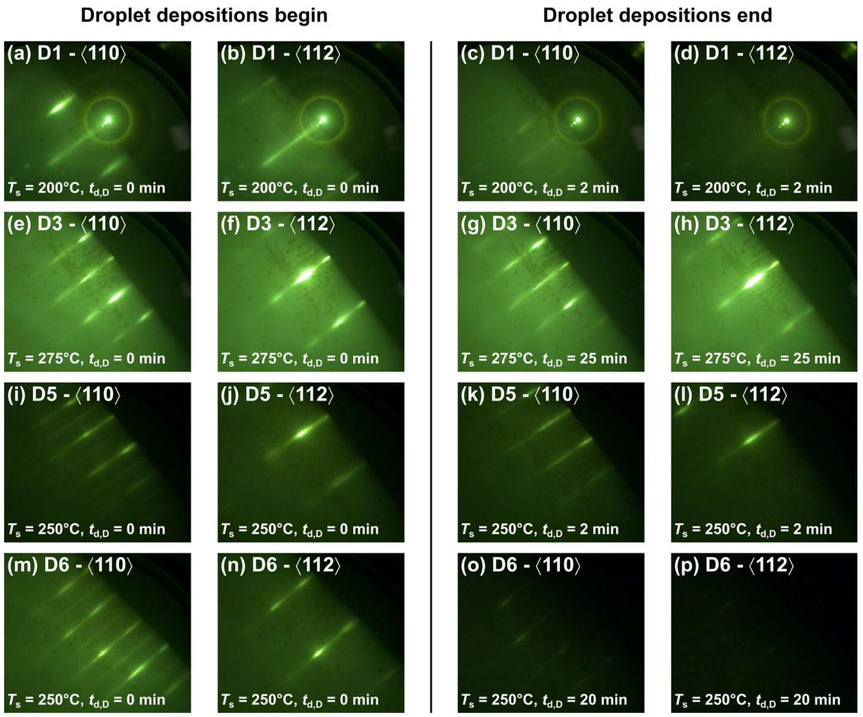

Figure S1. (a) and (b) RHEED patterns recorded along the 〈110〉 and 〈112〉 azimuths at the droplet deposition time ($t_{d, D}$) = 0 min for sample D1. (c) and (d) RHEED patterns recorded along the 〈110〉 and 〈112〉 azimuths at $t_{d, D}$ = 2 min for sample D1. (e) and (f) RHEED patterns recorded along the 〈110〉 and 〈112〉 azimuths at $t_{d, D}$ = 0 min for sample D3. (g) and (h) RHEED patterns recorded along the 〈110〉 and 〈112〉 azimuths at $t_{d, D}$ = 25 min for sample D3. (i) and (j) RHEED patterns recorded along the 〈110〉 and 〈112〉 azimuths at $t_{d, D}$ = 0 min for sample D5. (k) and (l) RHEED patterns recorded along the 〈110〉 and 〈112〉 azimuths at $t_{d, D}$ = 2 min for sample D5. (m) and (n) RHEED patterns recorded along the 〈110〉 and 〈112〉 azimuths at $t_{d, D}$ = 0 min for sample D6. (o) and (p) RHEED patterns recorded along the 〈110〉 and 〈112〉 azimuths at $t_{d, D}$ = 2 min for sample D6.

Figures S1 (a) and (b) show the reflection high-energy electron diffraction (RHEED) patterns of sample D1 recorded along the 〈110〉 and 〈112〉 azimuths at the droplet deposition time ($t_{d,\ D}$) = 0 min and the substrate temperature (T_s) = 200°C. The patterns diffracted from the GaAs (111) surface. After depositing Bi droplets for 2 mins, the diffraction patterns along the two azimuths of GaAs (111) dim, as depicted in Fig. S1 (c) and (d). The dimmed diffraction patterns confirm the droplet deposition that covers the GaAs surface.

Figures S1 (e) and (f) show the RHEED patterns of sample D3 recorded along the 〈110〉 and 〈112〉 azimuths at $t_{d,\ D}$ = 0 min, and T_s = 300°C. The diffraction patterns of GaAs (111) similar to those of Fig. S1 (a) and (b). (The discrepancy occurred from the position of the specular beam that differs from sample to sample.) After depositing Bi droplets for 25 mins, we cannot differentiate the diffraction patterns along the 〈110〉 and 〈112〉 azimuths (Fig. S1 (g) and (h)) from those before the droplet deposition (Fig. S1 (e) and (f)), suggesting the small amount of the deposited droplets at high T_s.

Figures S1 (i) and (j) show the RHEED patterns of D5 recorded along the 〈110〉 and 〈112〉 azimuths at $t_{d,\ D}$ = 0 min, and T_s = 250°C. After depositing Bi droplets for 2 mins, the diffraction patterns along the 〈110〉 and 〈112〉 azimuths dim, as depicted in Fig. S1 (k) and (l), confirms the droplet deposition on the surface. However, the RHEED intensity difference before and after the droplet depositions for this sample is not distinct compared to that of D1.

Figures S1 (m) and (n) show the RHEED patterns of D6 recorded along the 〈110〉 and 〈112〉 azimuths at $t_{d,\ D}$ = 0 min, and T_s = 250°C. After depositing Bi droplets for 20 mins, we can hardly see the diffraction patterns along the 〈110〉 and 〈112〉 azimuths (see Fig. S1 (o) and (p)), which confirms a large amount of the deposited droplets on the surface.

S2: Detailed TEM-EDS compositional measurements

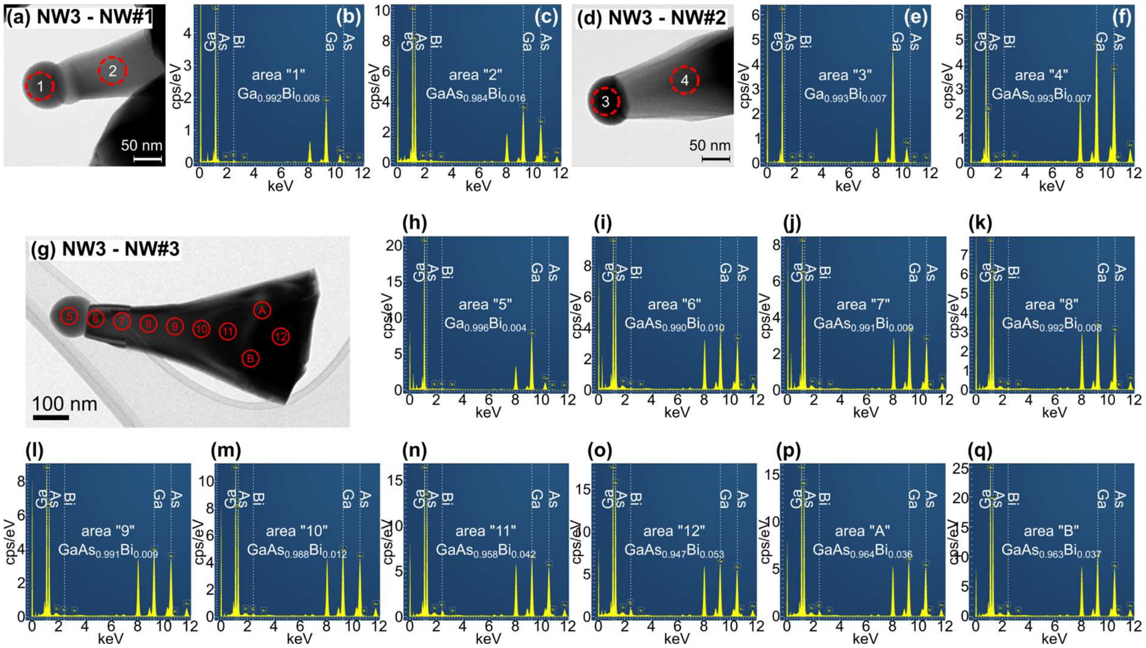

Figure S2. (a) BF-TEM micrograph of NW#1 taken from sample NW3. (b) and (c) Corresponding EDS spectra for NW#1 acquired at the droplet, area "1", and at the NW stem, area "2", respectively. (d) BF-TEM micrograph of NW#2 taken from sample NW3. (e) and (f) Corresponding EDS spectra for NW#1 acquired at the droplet, area "3", and at the NW stem, area "4", respectively. (g) BF-TEM micrograph of NW#3 taken from sample NW3. (h) – (q) Corresponding EDS spectra for NW#3 acquired at the droplet, area "5", and at the NW stem, areas "6", "7", "8", "9", "10", "11", "12", "A", and "B", respectively.

Figure S2 (a) depicts the bright-field (BF) transmission electron microscopy (TEM) image of nanowire#1 (NW#1) from sample NW3. Energy-dispersed X-ray spectroscopy (EDS) measurements were performed at the catalytic droplet (area "1") and the NW stem (area "2"). Their corresponding EDS spectra are shown in Fig. S2 (b) and (c), giving the compositions of $Ga_{0.992}Bi_{0.008}$ (at the droplet) and $GaAs_{0.984}Bi_{0.016}$ (at the NW stem). Figure S2(d) depicts the BF-TEM image of NW#2 from sample NW3. EDS measurements were performed at the catalytic droplet (area "3") and the NW stem (area "4"). Their corresponding EDS spectra are shown in Fig. S2 (e) and (f), giving the compositions of $Ga_{0.993}Bi_{0.007}$ and $GaAs_{0.993}Bi_{0.007}$, respectively. Figure S2 (g) depicts the BF-TEM image of NW#3 from sample NW3. EDS measurements were performed at the catalytic droplet (area "5") and the NW stem (areas "6", "7", "8", "9", "10", "11", "12", "A", and "B", consecutively showing the compositions of $Ga_{0.996}Bi_{0.004}$, $GaAs_{0.990}Bi_{0.010}$, $GaAs_{0.991}Bi_{0.009}$, $GaAs_{0.992}Bi_{0.008}$, $GaAs_{0.991}Bi_{0.009}$,

$GaAs_{0.988}Bi_{0.012}$, $GaAs_{0.958}Bi_{0.042}$, $GaAs_{0.947}Bi_{0.053}$, $GaAs_{0.964}Bi_{0.036}$, $GaAs_{0.963}Bi_{0.037}$. The compositions probed at the middle of the NWs vary from wire to wire with the Bi content in the 0.007 – 0.016 range. The studies on NW#3 show that with a stable substrate temperature, the incorporated Bi concentrations are stable both axially (see Fig. S2(h) – (l)) and across the wire diameter (see Fig. S2(p) and (q)). Note that the NW stem compositions are obtained after balancing the cation-anion stoichiometric condition.

S3: Top-view SEM images of GaP-passivated GaAsBi NWs (NW5): as-grown and scraped-out areas.

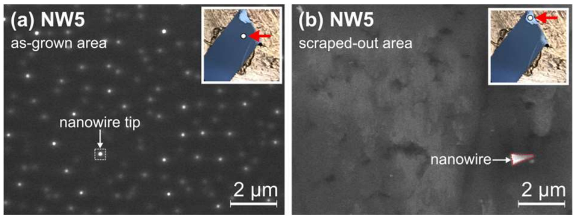

Figure S3. (a) and (b) are top-view SEM images of GaP-passivated GaAsBi nanowire (NW5) acquired at the as-grown and NWs-scraped-out area, respectively.

Figure S3 (a) shows the top-view SEM image of as-grown GaP-passivated GaAsBi NWs (sample NW5). Bright and dull white spots scattered in the image are the tips of the NWs, providing the NW density of 1.22 wire·μm^{-2}. We estimated the number of NWs (~1.5×10^5 wires) that contributed to the photoluminescence presented in the main text by considering the wire density and the laser spot diameter (~0.2 mm). Figure S3 (b) shows the top-view SEM image of sample NW5 after intentionally scraping out the NWs. There are no vertical NWs left on the surface, however, one can observe a horizontal NW attached to the surface during the scraped-out process. White dots in the insets of Fig. S3 (a) and (b) (pointed by the red arrows) are the positions on the sample where the SEM images were observed.
